# Easier removal of a gastrointestinal stromal tumor using a new detachable snare-assisted traction technique

**DOI:** 10.1055/a-2119-0508

**Published:** 2023-07-13

**Authors:** Zhang Tao, Jie Liu, Wen Feng Pu, Chao Lan, Ning Chuan Ren, Feng Ying Lin, Dan Hu

**Affiliations:** Department of Gastroenterology, Nanchong Central Hospital, The Second Clinical Medical College, North Sichuan Medical College, Nanchong City, Sichuan, China

A 51-year-old woman presented to our department with a stromal tumor (about 3 × 2 cm) in the gastric body ( Fig. 1 ). After the tumor had been marked, mucosal and submucosal incision was commenced, but it was not possible to make an effective incision owing to the narrow gap between the tumor and the normal muscularis propria caused by the effect of gravity on the tumor ( Video 1 ). A detachable snare was used to trap part of the tumor and a clip with a traction wire was used to clamp the detached snare to create traction. After traction had been applied via the detached snare, the incision gap was enlarged ( Fig. 2 ) and the tumor was then rapidly excised by the now effective incision ( Fig. 3 ). Pathologic examination showed that the tumor was a low risk gastrointestinal stromal tumor of 3 × 2 cm. Follow-up after 1 month showed that the wound had healed ( Fig. 4 ).

**Fig. 1:**
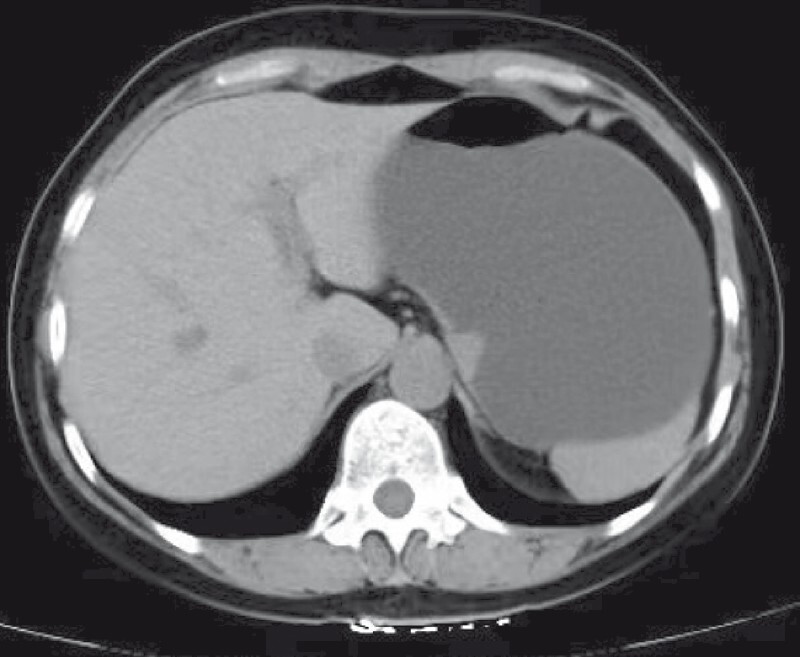
Computed tomography image showing a stromal tumor (about 3 × 2 cm) in the upper gastric body.

**Video 1** Easier removal of a gastrointestinal tumor is shown using a new detachable snare-assisted traction technique.


**Fig. 2:**
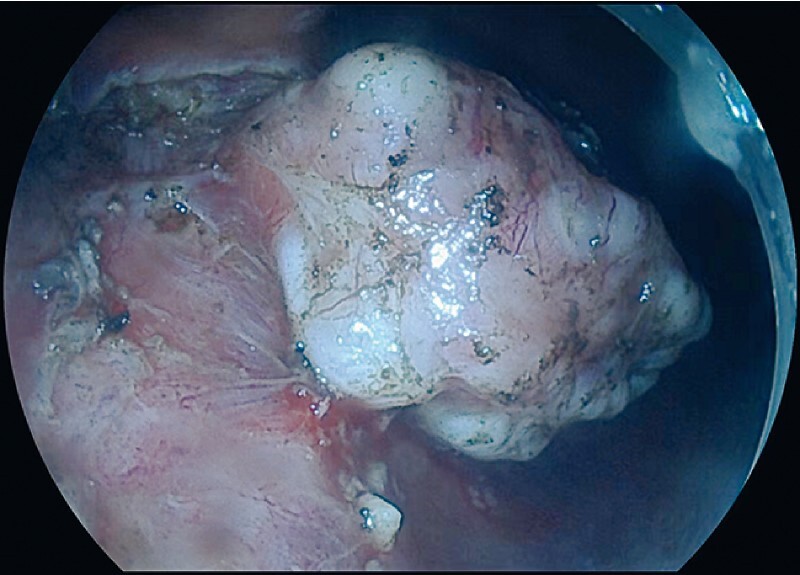
Endoscopic image showing the enlarged incision gap after traction had been applied via the detached snare.

**Fig. 3:**
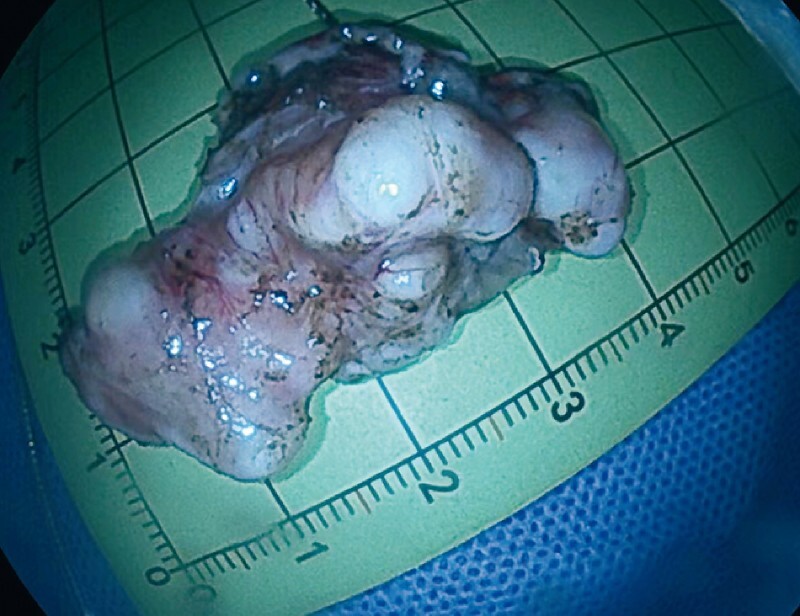
Macroscopic appearance of the resected tumor specimen.

**Fig. 4:**
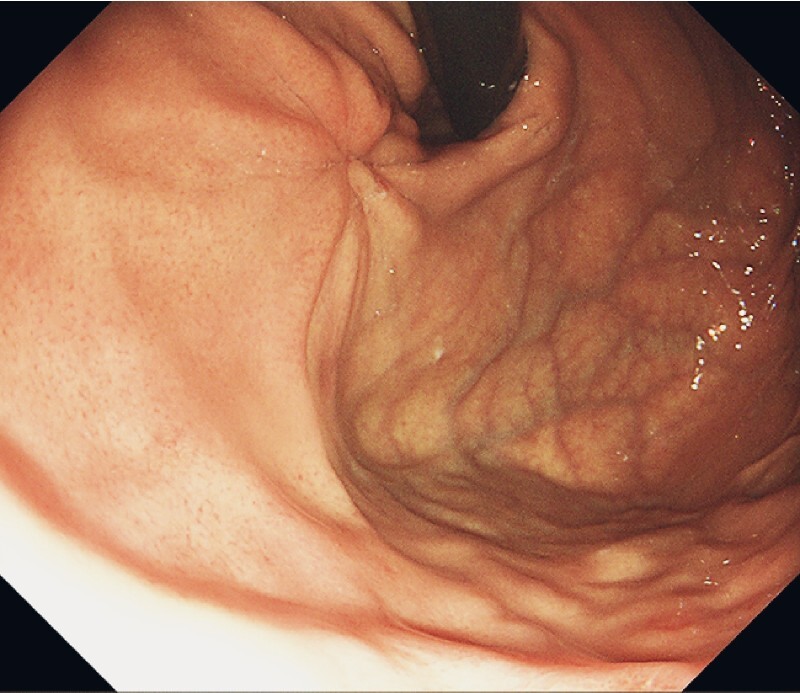
Follow-up endoscopy after 1 month showing the healed wound.

Traction techniques have effectively shortened the procedure time, and can help to avoid unnecessary bleeding and perforation during endoscopic submucosal dissection 1 2 . A clip with a traction wire has often been used to enlarge the incision gap 3 . For large tumors, snares are a useful traction method 4 ; however, the snare is hard to release when it has trapped the tumor. Therefore, we designed a detachable snare to trap the tumor and create traction that is then easier to release. This is first report of the use of the detachable snare to create effective traction to remove a tumor.

Endoscopy_UCTN_Code_CCL_1AB_2AD_3AB

## Notes

Endoscopy E-Videos

https://eref.thieme.de/e-videos
*E-Videos* is an open access online section of the journal *Endoscopy* , reporting on interesting cases and new techniques in gastroenterological endoscopy. All papers include a high-quality video and are published with a Creative Commons CC-BY license. Endoscopy E-Videos qualify for HINARI discounts and waivers and eligibility is automatically checked during the submission process. We grant 100% waivers to articles whose corresponding authors are based in Group A countries and 50% waivers to those who are based in Group B countries as classified by Research4Life (see: https://www.research4life.org/access/eligibility/ ). This section has its own submission website at https://mc.manuscriptcentral.com/e-videos
